# Comparative Effect of Self- or Dual-Curing on Polymerization Kinetics and Mechanical Properties in a Novel, Dental-Resin-Based Composite with Alkaline Filler. Running Title: Resin-Composites with Alkaline Fillers

**DOI:** 10.3390/ma11010108

**Published:** 2018-01-11

**Authors:** Nicoleta Ilie

**Affiliations:** Department of Conservative Dentistry and Periodontology, University Hospital, LMU Munich, Goethestr 70, 80336 Munich, Germany; nilie@dent.med.uni-muenchen.de; Tel.: +49-89-44005-9412; Fax: +49-89-44005-9302

**Keywords:** resin-composite, alkaline fillers, degree of cure, polymerization kinetics, bulk fill, resin based composite

## Abstract

Dental bulk-fill restorations with resin-composites (RBC) are increasing in popularity, but doubts concerning insufficient curing in depth still disconcert clinicians. An alternative might be offered by modern dual-cured RBCs, which additionally provide bioactive properties. This study assessed the impact of additional light-curing on polymerization kinetics, the degree of conversion (DC) and mechanical properties of a novel, dual-cured RBC with alkaline fillers. Since the bioactivity of a material often implies a release of compounds, the mechanical stability in simulated clinical environments was also evaluated. Polymerization kinetics and DC were assessed at 2- and 4-mm specimen depths in real-time up to one hour (*n* = 6). Incident and transmitted irradiance and radiant exposure were recorded at 2- and 4-mm depths. Micro-mechanical profiles (*n* = 6) were assessed in 100-µm steps along 6-mm deep specimens at 24 h post-polymerization. Flexural strength and modulus (*n* = 10) were determined up to three months of immersion in neutral (6.8) and acidic (4) pH conditions. DC variation in time was best described by a sigmoidal function (R^2^ > 0.98), revealing a retarded (3.4 ± 0.4 min) initiation in C=C double bond conversion in self-cured versus dual-cured specimens. The setting reaction kinetic was identical at 2- and 4-mm depths for the self-cure mode. For the dual-cure mode, polymerization initiated at 2-mm depth instantly with light-irradiation, while being retarded (0.8 min) at 4-mm depth. The material behaves similarly, irrespective of curing mode or depth, later than 11 min after mixing. Flexural strength and modulus was comparable to regular RBCs and maintained up to three months in both neutral and acidic conditions. Additional light-curing initially accelerates the polymerization kinetic and might help shorten the restauration procedure by hardening the material on demand, however with no effect on the final properties.

## 1. Introduction

The demand for a faster and simplified restorative procedure led to the development of bulk-fill RBCs that are able to be cured in increments up to 4 or 5 mm thickness [1,2]. An enhanced depth of cure challenges and limits the choice of RBC constituent parts, since it requires an optimized filler/resin refractive index mismatch [3] to enhance light transmittance, which is dependent on the chemical composition of both filler and matrix. Apart from a new photo-initiator implemented in one material [4], the identified mechanisms to enhance the depth of cure in light-cured bulk-fill RBCs are the reduction of the filler–matrix interface by enlarging the filler size [5] and the decrease in the amount of pigments. Beyond fast processing and adequate curing in large increments, modern bulk-fill RBCs require additional features to make them more performant and be accepted, such as i.e., bioactivity or self-adhesiveness. Within the light-cured bulk-fill RBCs, only giomers [6] are recognized as releasing ions such as F^−^, Na^+^, Sr^2+^, Al^3+^, BO_3_^3−^, SiO_3_^2−^ [7] able to assure a mineralizing and cariostatic potential. This feature is accounted to the pre-reacted glass-ionomer (PRG) fillers [8], made of a fluoroaluminosilicate glass that has been reacted with a polyalkenoic acid in the presence of water.

The increasing acceptance for a fast restorative procedure with light-cured, bulk-fill RBCs is still overshadowed by the uncertainty of an adequate curing in depth [9]. This aspect as well as the attempt to offer a valuable, aesthetic, basic filling material might have been motivations for the recent launch on the market of several dual-curing RBCs that are also suitable for a bulk-filling procedure. In addition, these materials focus on bioactive properties and are designed to release acid-neutralizing ions to prevent tooth demineralisation. For this purpose, one approach used alkaline fillers that are implemented in a methacrylate resin matrix, advertised as a new material category—the alkasites [10].

Dual-curing RBCs consist in general of two components that have to be mixed together, each containing a different initiator system. Thus far, dual-cure RBCs have been mainly used in modern dentistry for core build-up and cementation. An often raised question in this context was whether the self-curing polymerisation reaction is sufficient in the event of impeded light transmittance, or even in the absence of light. A comparison of the properties of dual-cure RBCs that were either self-cured or additionally exposed to various light irradiation protocols showed that the effect of light irradiation is material dependent, providing from no impact to a high impact on the final material properties [11]. 

Therefore, the main factors to be considered for the novel dual-cured RBC with alkaline fillers, apart from the caries protective ability, are the length of its setting time, the sufficiency of its mechanical properties and degree of conversion in depth, as well as the impact of additional light-curing on these properties. Compared to light-initiated polymerization, which allows curing a material on demand, a redox activation of the polymerisation process occurs slower and might not meet modern clinical requirements for a fast restorative procedure. Therefore the setting time of the analyzed material should be known and adjusted within the regular clinical treatment duration, also in the absence of light. Moreover, the presence of alkaline fillers with a higher refractive index compared to regular silicate glass fillers might jeopardize the filler/resin refractive index match that is necessary for enhanced light transmission in depth. The benefit would be a more opaque material, able to mask the dark visual effect of the oral cavity or the tooth’s structural discolorations more efficiently than several translucent, light-cured, bulk-fill RBCs. Whether the redox activation is able to compensate for the lower amount of light reaching deeper areas of a restoration, avoiding a gradation in mechanical properties in depth must be verified. The null hypotheses tested were that: (a) the curing mode (self or dual) has no effect on polymerisation kinetics, degree of conversion (DC) and mechanical properties; (b) specimen depth has no effect on DC and mechanical properties and (c) immersion media and duration have no effect on the mechanical properties of the RBC with alkaline fillers.

## 2. Materials and Methods

An RBC with alkaline fillers (Cention N, Ivoclar Vivadent, Schaan, Liechtenstein, LOT U19921, A2) was set to either self or dual. The two components of the material were used by mixing one scoop (included in the kit) of powder with one drop of liquid, thus corresponding to a powder:liquid ration of 4.6:1 *w*/*w*. 

The degree of conversion and polymerisation kinetics parameters were assessed in real-time for one hour at clinically-relevant increment thicknesses (2-mm and 4-mm). Besides, incident and transmitted light through the above-mentioned specimen geometries were recorded. Micro-mechanical properties were evaluated in 100-µm steps in a line profile along 6-mm high specimens, after storing them in water at 37 °C for 24 h. Additionally, the effect of the immersion medium (acidic or neutral buffer solution at 37 °C) and immersion duration (24 h, six weeks and three months) was evaluated by means of flexural strength and modulus. In tests that additionally assessed the effect of light-irradiation, the LED light curing unit Bluephase N (Ivoclar Vivadent, Schaan, Liechtenstein) was used in the high power program and for an exposure time of 20 s. 

### 2.1. Degree of Conversion (DC)

The DC of the investigated material was measured using Fourier transform infrared (FTIR) spectroscopy with an FTIR spectrometer fitted with an attenuated total reflectance (ATR) accessory (Nicolet iS50, Thermo Fisher, Madison, WI, USA). Profiling was performed in real-time over 60 min taking one spectra each of 0.4 s using two distinct specimen geometries at specimen depths of 2 and 4 mm. Teflon specimen molds (3 mm diameter, 2 and 4-mm thick) were filled in bulk with the RBC under investigation. Therefore powder and liquid were mixed by daylight within 15 s, than applied to the molds, while the FTIR records started 30 s after initiating the mixture procedure. For the investigated groups (two setting modes and two specimen depths), six samples were employed to determine DC. The non-polymerized material was applied directly onto the diamond ATR crystal in the respective molds and covered with a transparent matrix strip. The material was either light irradiated for 20 s or self-cured and DC was measured at the bottom surface of the specimens. DC was calculated by assessing the variation in peak height of the absorbance intensity of the methacrylate carbon-to-carbon (C=C) double bond peak at 1634 cm^−1^ using Equation (1).

(1) $$\mathrm{DC}\%\;=\;[1\;-\;\frac{{\left(1634{\mathrm{cm}}^{-1}\right)}_{\mathrm{Peak}\;\mathrm{height}\;\mathrm{after}\;\mathrm{curing}}}{{\left(1634{\mathrm{cm}}^{-1}\right)}_{\mathrm{Peak}\;\mathrm{height}\;\mathrm{before}\;\mathrm{curing}}}]\;\times \;100$$

A sigmoidal model (Hill, 3 Parameter) was considered to describe the variation of DC in time. The correlation function is outlined in Equation (2).

(2) $$y=A\frac{x^{b}}{c^{b}+x^{b}}$$

where the parameters *A*, *b* and *c* were the modulation factors of the sigmoidal function to optimize the sigmoidal function on the measured curve plotted on a DC versus time.

### 2.2. Light Transmittance

Specimens were prepared in cylindrical Teflon molds (6 mm diameter, increment thickness 2 mm and 4 mm, *n* = 3), and cured by applying the aforementioned curing unit directly, perpendicular and centered on the sample’s surface using a mechanic arm. The exposure time was 20 s. While the specimens were cured, a laboratory-grade National Institute of Standards and Technology (NIST)-referenced USB4000 Spectrometer (MARC (Managing Accurate Resin Curing) System, Blue light Analytics Inc., Halifax, NS, Canada) measured in real-time the irradiance at the bottom of the specimens. The cylindrical Teflon molds containing the material were aligned centered on the round detector of the spectrometer, which had a diameter of 3.9 mm. Consequently, the irradiance and radiant exposure reaching this area was considered. The miniature fiber optic USB4000 Spectrometer employs a 3648-element Toshiba linear Charge-coupled Device (CCD) array detector and high-speed electronics (Ocean optic, Largo, FL, USA). The spectrometer was calibrated using an Ocean Optics’ NIST-traceable light source (300–1050 nm). The system uses a CC3-UV Cosine Corrector (Ocean optic, Largo, FL, USA) to collect radiation over a 180° field of view, thus mitigating the effects of optical interference associated with light collection sampling geometry.

Irradiance and radiant exposure at a wavelength range of 360–540 nm were individually collected at a rate of 16 records/s. The sensor was triggered at 20 mW. The radiant exposure was calculated by integrating the irradiance versus the wavelength at the used exposure time (20 s). In addition, the incident radiant exposure (the radiant exposure reaching the specimen’s surface) was determined on three occasions using the above-described spectrometer, by applying the curing unit directly to the sensor. 

### 2.3. Micro-Mechanical Properties Profiles

Cylindrical specimens (height = 6 mm, diameter = 10 mm) were prepared in cylindrical metal molds. Therefore, the material was used either as self-cured or dual-cured. For the self-cured condition, the material was applied to the metal mold and allowed to set for 4 min. Specimens were then removed from the mold and stored in 37 °C distilled water for 24 h (*n* = 6). For the dual-cure condition (*n* = 6), specimens were polymerized for 20 s and stored after 4 min in 37 °C distilled water for 24 h. Prior to measurements, specimens were sectioned in the middle with a slow-speed diamond saw (Isomet low-speed saw, Buehler, Germany) under water cooling, and polished with a diamond suspension (mean grain size: 1 µm). Measurements were made with an automatic micro-hardness indenter (Fischerscope H100C, Fischer, Sindelfingen, Germany) starting at 0.1 mm under the surface, with 100-µm intervals between the measuring points, with a total of 360 single indentations for each curing condition. 

The test procedure was carried out in a force-controlled manner, where the test load increased and decreased with constant speed between 0.4 mN and 500 mN. The load and penetration depth of the indenter were continuously measured during the load–unload hysteresis. Universal hardness is defined as the test force divided by the apparent area of indentation under applied test force. From the multiplicity of measurements stored in a database supplied by the manufacturer, a conversion factor between universal hardness and Vickers hardness (HV) was calculated and inputted into the software. The indentation modulus (Y_HU_) was calculated from the slope of the tangent of the indentation depth curve at maximum force. The hardness and indentation modulus variation with depth were calculated for each group based on data from six samples.

The depth of cure (DOC), usually acknowledged as the thickness of an RBC that is adequately cured or rather as the depth where the HV equals the surface value multiplied by an arbitrary ratio, usually 0.8 [12] (=HV-80%) was also calculated. Therefore, for each sample, the HV in depth is compared to the surface value at the most favorable curing conditions, and noted when it became less than 80%. This depth is defined as DOC. 

### 2.4. Flexural Strength (FS) and Flexural Modulus (FM)

The flexural strength and flexural modulus were determined in a three-point-bending test (*n* = 10) in analogy to ISO 4049:2009. Therefore, 60 samples were made by compressing the material between two glass plates with intermediate Polyacetate sheets, separated by a Teflon mold with internal dimensions of 2 × 2 × 18 mm^3^. The material was pressed in a clip and stored in the mold for one hour at 37 °C in a water-saturated atmosphere. Thereafter, specimens were removed from the mold and then stored either in acidic (pH = 4, lactate) or neutral (pH = 6.8, phosphate) buffer solution at 37 °C prior to testing for 24 h, six weeks or three months. The specimens were loaded until failure in the universal testing machine (Zwick/Roell Z 2.5, AST. GmbH, Ulm, Germany). The crosshead speed was 0.5 mm/min. The three-point bending test device is constructed according to the guidelines of NIST No. 4877 with 12 mm distance between the supports. During testing the specimens were immersed in distilled water at room temperature. 

### 2.5. Statistical Analyses

A Shapiro–Wilk test verified the normal distribution of the data. A multivariate analysis (general linear model) assessed the effects of various parameters as well as their interaction terms on DC, micro-mechanical properties, as well as flexural strength and flexural modulus. The partial eta-squared statistic reported the practical significance of each term, based on the ratio of the variation attributed by the effect. Larger partial eta-squared (η_P_^2^) values indicate a greater amount of variation accounted for by the model, which figure up to a maximum of 1.0. One-way ANOVA was additionally performed for the various measured parameters. In all statistical tests, *p*-values (the probability for the statistical model that, when the null hypothesis is true, the sample mean difference between two compared groups is the same as or of greater magnitude than the actual observed results) < 0.05 were considered statistically significant using SPSS Inc. (Version 23.0, Chicago, IL, USA).

## 3. Results

### 3.1. Degree of Conversion (DC)

**Figure 1:**
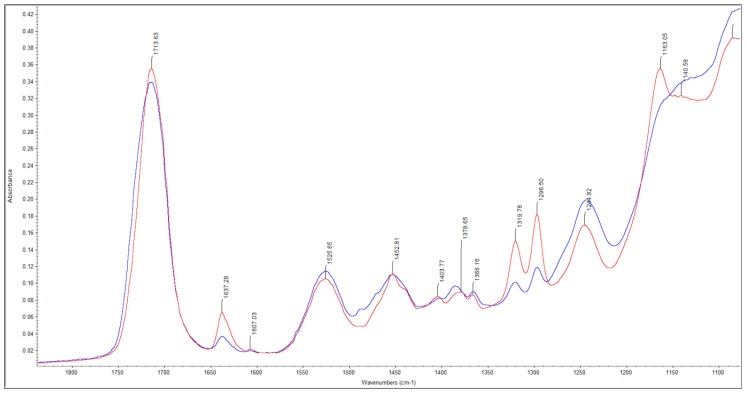
FTIR spectra of an RBC with alkaline fillers recorded immediately after mixing (red curve) as well as one hour after setting (blue curve) in the self-cure mode.

All analyzed parameters—*curing mode*, *specimen depth* and *observation time*—as well as their binary and ternary interaction products exerted a significant (*p* < 0.001) effect on DC. The highest influence was exerted by the parameter *observation time* (*p* < 0.001; η_P_^2^ = 0.978), followed by the parameter *curing mode* (*p* < 0.001; η_P_^2^ = 0.431), while the influence of the parameter *specimen depth* was extremely low (*p* < 0.001; η_P_^2^ = 0.086). From all binary interaction products, the highest effect was identified for the combination *curing mode* × *observation time* (*p* < 0.001; η_P_^2^ = 0.519). Figure 1 presents an example IR spectrum for the set and freshly-mixed material. 

**Figure 2:**
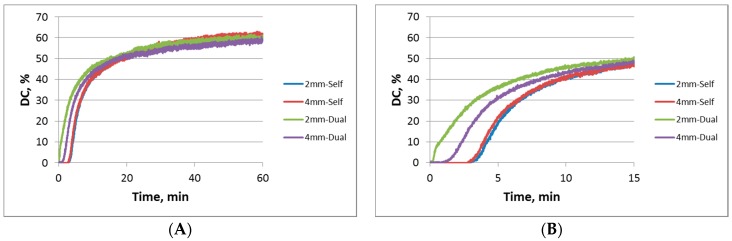
Degree of conversion variation (mean values, *n* = 6) as a function of curing mode and specimen depth; (**A**) observation time of one hour; (**B**) close-up view of the first 15 min of observation.

The variation of DC in time as a function of curing mode and specimen depth is presented in Figure 2A and was best described by a sigmoidal function, manifested by a high correlation factor of R^2^ > 0.98 defined for the parameters A, b and c (Table 1). Figure 2B is a close-up view of the first 15 min of observation, revealing a retarded (3.4 ± 0.4 min) initiation in C=C double bond conversion in the self-cured compared to the dual-cured specimens.

The kinetic of the setting reaction is identical at 2 and 4 mm depth for the self-cure mode. In contrast, the polymerization initiates shortly after light-irradiation in the dual-cure mode at a depth of 2 mm, while being retarded (0.8 min) for the same curing mode at 4 mm depths. 

The DC mean values determined as a function of *curing mode* and *specimen depth* vs. time are summarized in Table 2 at representative observation times. 

Since a high number of spectra (9140) were collected within one hour of observation in each specimen, one-way ANOVA was performed at selected time intervals that were gathered in a sequence of 0.1 min in the first minute of observation, and continued with a sequence of 0.5 min until the end of the experiment. Table 2 identifies the time intervals where changes in the statistical relation among DC values at different curing conditions and depths occurred. Starting with 0.4 min after mixing and up to 2.5 min, significantly higher DC values were measured for the 2-mm, dual-cure group compared to all other analyzed conditions. At 2.5 min, DC_2mm Dual-cure_ was still significant higher, followed by DC_4mm Dual-cure_, while in the self-cured specimens the polymerization had not yet initiated. Starting with an observation time of 3.5 min, DC_2mm Dual-cure_ and DC_4mm Dual-cure_ were statistically similar and higher compared to the self-cured groups. This statistical relation among groups remains valid up to 10.5 min. Starting with 11 min and up to one hour, no significant differences were registered among groups. 

**Figure 3:**
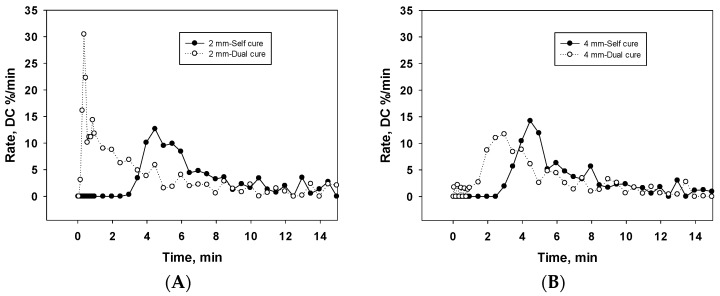
Rate of carbon–carbon double-bond conversion per minute within the first 15 min of setting at a depth of: (**A**) 2 mm and (**B**) 4 mm.

The rate of C=C double bond conversion (Figure 3A,B) reveals similar behavior for 2 and 4 mm self-cured specimens, while the largest rate of polymerization (13.5 ± 1.1%/min) was identified at 4.5 min. 

The rate curve measured at a depth of 4 mm in the dual-cured mode was similar in shape to the curve identified in self-cured specimens, but was shifted to the left, to shorter time values. The rate maximum was identified at 2.5 min. In contrast, the highest rate within all setting conditions (30.5%/min) was identified in dual-cured specimens at a depth of 2 mm, at a very early observation time.

### 3.2. Light Transmittance

**Figure 4:**
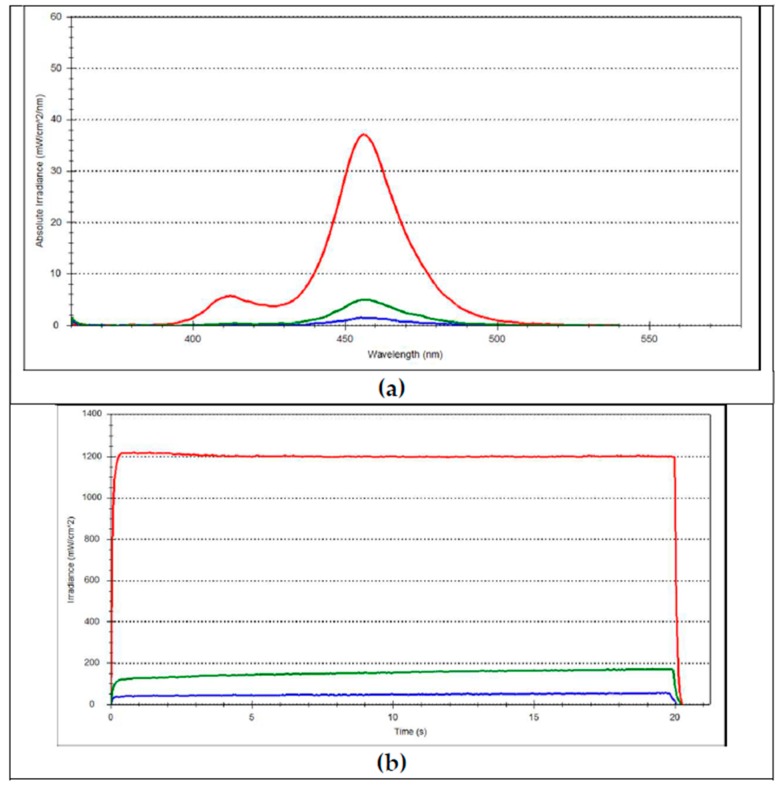
(**a**) Emission light spectrum of the used LCU (red) and transmitted spectrum through 2-mm (green) and 4-mm (blue) increments of the RBC with alkaline filler. (**b**) Variation of irradiance in time for the same conditions.

The incident irradiance was determined as (1306.6 ± 12) mW/cm^2^, while the incident radiant exposure for an exposure time of 20 s was (25.0 ± 0.5) J/cm^2^. The spectral distribution revealed two peaks, identified at 456 nm and 412 nm. A consistent drop in transmitted light related to the incident light was observed at both 2 mm ((166.5 ± 8.6) mW/cm^2^ and (3.0 ± 0.1) J/cm^2^) and 4 mm ((37.8 ± 2.9) mW/cm^2^ and (0.6 ± 0.1) J/cm^2^) (Figure 4). A slow but progressive enhanced light transmittance during polymerization was identified in both 2-mm and 4-mm specimens (Figure 4). 

### 3.3. Micro-Mechanical Property Profiles

For both curing conditions, the HV in depth was higher than the 80% value measured at a sub-surface of 0.1 mm. Therefore, the depth of the cure under the measured conditions was 6 mm in all specimens. 

For statistical analysis of the variation in mechanical properties with depth, data recorded within a 0.5 mm depth interval was grouped together. No significant change in the measured micro-mechanical properties with depth was identified for both self- and dual-cured groups (Table 3). A multivariate analysis revealed that the curing mode did not influence the material’s properties (*p* = 0.187 for HV, 0.254 for Y_HU_ and 0.168 for the Martens hardness). 

### 3.4. Flexural Strength and Modulus

**Figure 5:**
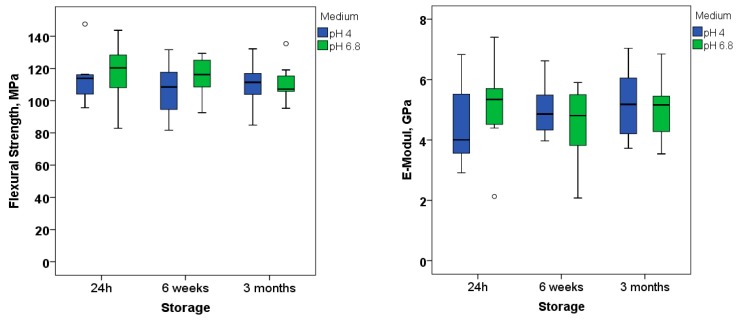
Flexural strength (**left**) and flexural modulus (**right**) at different immersion times in neutral (6.8) or acidic (4) pH.

Neither the immersion medium nor the immersion time or their interaction product influenced the flexural strength and modulus (Figure 5; *p*-values are summarized in Table 4).

## 4. Discussion

The fundamental approach of this study was to evaluate the impact of curing mode on the polymerization kinetics, degree of conversion and mechanical properties of a novel RBC with alkaline fillers for basic filling purposes. 

The analyzed material is designed as a self-curing material with optional light-curing perspectives. It consists in its current form of two components—a fluid and a powder—that have to be mixed together, but attempts to improve handling by means of a capsulated system are in progress. While the initiation rates can be very fast in the light-curing mode (Figure 3), its significant limitation is that the penetration of light energy in depth is low, as identified in Figure 4. In support of this, a redox activation was added consisting of a copper salt, a peroxide and a thiocarbamide and was implemented in the powder component [10]. The kinetics of redox-initiated polymerization often proceed similarly to light-initiated polymerization in terms of propagation and termination steps. For self-curing, both DC and kinetics were not influenced by depth. As identified in the DC variation in time, the material started to set at 3.4 ± 0.4 min after mixing the fluid with the powder. This time interval may be accordingly noted as the working time. Up to this point, the C=C double bond conversion starts to increase, first at a low rate, then increasingly faster, culminating at 4.5 min with a rate of 13.5 ± 1.1%/min. 

In contrast, for light-curing, two Norrish Type 1 initiators—a dibenzoyl germanium derivative: bis-(4-methoxybenzoyl)diethylgermane) and an acyl phosphine oxide (BAPO (Irgacure 819), bis(2,4,6-trimethylbenzoyl) phenylphosphine oxide)—were added to the liquid component [10]. Both initiators might have been efficiently activated under the applied curing conditions, since a violet-blue LED light curing unit (LCU) (λ_max violet_ = 412 nm, λ_max blue_ = 456 nm, Figure 4) was employed for polymerization, with an emission spectrum in the wavelength range of 385–515 nm. Moszner et al. described two germanium-based photo-initiators—BTMGe and DBDEGe—with a lower absorption maximum (λ_max_ = 411 nm and λ_max_ = 418 nm, respectively) when compared to Camphorquinone (CQ, λ_max_ = 468 nm) [13]. They match well with the emission spectrum (Figure 4) of the used LCU that reveals a maximum peak at 412 nm in the violet wavelength range. It must however be considered that the absorption spectra of the germanium-based photo-initiators extend up to 460 nm, thus the initiator might also be partially activated by the blue wavelength range of the used LCU. Besides matching the emission spectrum of the LCU, the reactivity of the germanium-based photo-initiators was identified to be higher compared to CQ, a fact attributed to their larger molar extinction coefficient (ελ) [13]. A large ελ indicates a high probability of light absorption at a certain wavelength, leading to large quantum yields of the initiating species and improved degrees of conversion. For the germanium-based initiators, a significantly stronger absorption at their maximum was identified (BTMGe, ε = 1460 dm^2^ mol^−1^ and DBDEGe, ε = 5470 dm^2^ mol^−1^) in comparison to CQ (ε = 380 dm^2^ mol^−1^) [13]. BAPO, in contrast, initiates in the violet wavelength range with absorption peaks located at 295 nm and 370 nm [14]. Similarly to the germanium-based initiator, BAPO is a more efficient initiator when compared to CQ, since it undergoes homolytic α-cleavage of the C–P bond and generates two free radical species, i.e., [–(O=)C•] and [•P(=O)<], both capable of initiating polymerization [14].

Albeit their being very efficient, both light-curing initiators are activated at shorter wavelengths compared to CQ. The attenuation of light in depth is known to follow an exponential decay (Lambert’s law) [15], and is wavelength dependent. Hence, the violet light, characterized by shorter wavelengths, will be attenuated faster in depth as the blue light. This is clearly confirmed by the light transmittance measurements, since the transmitted violet light was below the sensitivity limit of the spectrometer at 4 mm depths, and was barely perceivable at 2-mm depths. It should also be noted that less than 10% of the incident irradiance was transmitted through 2-mm increments while less than 3% reached the bottom of the 4-mm increments. Accordingly, the light transmittance of the analyzed material is comparable with values measured in regular RBCs [16], whereas compared to light-cured, bulk-fill RBCs, the material is more opaque [5,16]. The latter may be an advantage for improving aesthetics, since several light-cured, bulk-fill RBCs, owing to their high translucency, are not able to efficiently mask the dark visual effect of the oral cavity or a tooth’s structural discolorations. The lower translucency of the analyzed material may be related to the used alkaline fillers that induce a higher filler/resin refractive index mismatch [17]. The decrease in light transmittance with depth is indeed evident in the kinetics of polymerization that reveal a retarded start of polymerization at a depth of 4 mm, compared to 2 mm, in light-activated specimens. In contrast, this aspect is less evident in light-cured, bulk-fill RBCs, in which the maximal rate of carbon–carbon double-bond conversion was identified to be independent of specimen depth [5]. A certain contribution of light to the polymerization process at 4-mm depths cannot be excluded, since the polymerization initiates in a less retarded manner when compared to the self-cure mode. This contribution must be attributed to the germanium-based initiator that is partially activated by blue light, as discussed above. Disregarding the retarded initiation, the DC curves vs. time reveal a similar shape in the 4-mm dual-cured group and in both self-cured groups, suggesting that the effect of light-curing is indeed low. In contrast, polymerization initiates instantly with light irradiation in the 2-mm dual-cured specimens, revealing a break in the variation of DC vs. time as soon as light irradiation terminates. It could therefore be shown that self and dual-curing compete only in the initial stage of polymerization, while the DC vs. time curves are perfectly superposed in all analyzed groups at 11 min after mixing. An addition, light-curing might in fact not improve the DC later than 11 min, but might contribute to an improved clinical applicability of the material, since the material can be set on demand. The measured DC data vs. time indicates that the curves do not lead to a plateau up to one hour of observation, thus DC might continue to an increase in time in all specimens. This is in accordance with previous observations, since in light-cured RBCs post-polymerization is identified that may extend, depending on the material, from one to 24 h after irradiation [18]. Accordingly, the post-polymerization was assessed in the present study by the measurement of the micro-mechanical properties [18] (hardness, indentation modulus) at 24 h post-curing. While the DC continues to increase, potential differences in the DC within the analyzed groups above one hour are, however, not plausible. This statement is sustained by the measured micro-mechanical properties, confirming a similar material performance in both the analyzed depths and curing modes. Accordingly, the curing mode (self or dual) has a significant effect on the polymerization kinetics and degree of conversion (DC), however only within the first 11 min of setting, but has no influence on the micro-mechanical properties, thus allowing the rejection of the first null hypothesis. Since the micro-mechanical properties are similar irrespective of depth, but DC depends on depth in dual-cured species within the first minutes after irradiation, the second null hypothesis is also rejected. The organic matrix of the analyzed material is described as containing Urethane dimethacrylates, either aliphatic (UDMA) or aromatic-aliphatic (Tetramethyl-xylylendiurethane dimethacrylate), as well as low viscosity dimethacrylates such as Tricyclodecan-dimethanol Dimethacrylate (DCP) and Polyethylene glycol 400 dimethacrylate (PEG-400 DMA) [10]. Since the material is Bis-GMA free, but contains a certain amount of an aromatic-UDMA, the peak at 1608 cm^−1^ that corresponds to the aromatic C=C double bond is detectable, as noticeable in Figure 1, but is very small, and therefore not precise enough to be taken as an internal standard for calculating DC. In real-time DC measurements, however, a reference peak can be neglected [19].

As for the filler system, the material contains 78.4% by weight inorganic fillers (Ba-Al-; Ca-Ba-Al-F- and Ca-F-Silicate glass as well as YtF_3_ and other customized fillers) [10]. The inorganic filler amount is directly related to the mechanical properties [20]. Neither an influence of the immersion medium nor of the immersion time was identified. At three months of immersion in a neutral buffered solution (pH = 6.8), the flexural strength and modulus reached a mean value of 110.5 ± 10.8 MPa and 5.0 ± 1.0 GPa, while amounting to 110.5 ± 13.4 MPa and 5.03 ± 1.1 GPa in an acidic medium (pH = 4). These values are within the range of data recorded for nano- and micro-hybrid RBCs [20]. Considering that the RBC with alkaline fillers was analyzed in the present study from a bulk-fill perspective, a direct comparison with low and high viscosity bulk-fill RBCs seems pertinent. Based on internal data measured under similar conditions, the analyzed material performed in a statistically similar manner with regard to the flexural strength to the bulk-fill RBCs Beautifil Bulk restorative (106.0 ± 12.7 MPa), Beautifil Bulk Flow (120.1 ± 8.2 MPa), Tetric EvoCeram Bulk Fill (120.8 ± 12.7 MPa), Filtek Bulk Fill (122.4 ± 9.6 MPa), Tetric EvoFlow Bulk Fill (123.5 ± 5.7 MPa) and Venus Bulk Fill (124.2 ± 12.1 MPa), while performing in an inferior manner compared to the materials SureFil^®^ SDR™ flow (130.5 ± 11.0 MPa), X-tra Fil (137.0 ± 14.4 MPa), QuixFil (138.6 ± 20.5 MPa), Filtek™ Bulk Fill Flowable Restorative (139.0 ± 3.5 MPa), x-tra base (139.4 ± 7.0 MPa) or SonicFill (142.8 ± 12.9 MPa).

The analyzed material is described as releasing fluoride (F^−^), hydroxide (OH^−^) and calcium (Ca^2+^) ions [10] able to prevent tooth demineralization. The mechanical performance of RBCs containing bioactive fillers is perceived as controversial in the literature, thus being identified either as stable [21] or instable in time [22]. Besides, the critical pH of dental caries is well established to be in the range of 5.5–5.7 [23], pH levels below this threshold will initiate the dissolution of enamel. Therefore, the mechanical stability of the RBC was analyzed in both neutral and acidic buffered solutions and was identified to be stable up to three months, allowing the acceptance of the third null hypothesis. 

## 5. Conclusions

The novel RBCs with alkaline fillers behave similarly at observation times above 11 min after mixing, irrespective of curing mode and depth, as confirmed by the DC and micro-mechanical properties. Additional light-curing only accelerates the polymerization kinetic initially, helping to shortening the restauration procedure by hardening the material on demand. Light transmittance through 2 and 4 mm thick increments is comparable to regular RBCs and lower compared to light-cured, bulk-fill RBCs. The flexural strength and modulus were comparable to regular RBCs and were maintained up to three months in both neutral and acidic conditions. The latter suggest good mechanical stability.

## Figures and Tables

**Table 1:** Polymerization kinetic parameters of the sigmoidal function.

Curing Mode	Width, mm	R^2^	A	b	c
self	2	0.99	59.5	2.1	7.4
	4	0.98	61.8	1.8	7.6
dual	2	0.98	65.2	0.9	3.9
	4	0.99	58.4	1.5	5.1

**Table 2:** Descriptive statistic (mean and standard deviation, SD) for the DC (%) at various observation times as function of curing mode and incremental thickness. Superscripts (a, b, c) indicate statistically homogeneous subgroups within a line (Tukey’s HSD (honest significant difference) test, α = 0.05).

Time, min	2 mm Self-Cure		4 mm Self-Cure		2 mm Dual-Cure		4 mm Dual-Cure	
	Mean	SD	Mean	SD	Mean	SD	Mean	SD
0.05	0.0	0.0	0.0	0.0	0.0	0.0	0.1	0.2
0.4	0.0 ^a^	0.0	0.0 ^a^	0.0	5.0 ^b^	4.5	0.2 ^a^	0.6
2.5	0.0 ^a^	0.0	0.0 ^a^	0.0	24.4 ^c^	6.8	12.5 ^b^	7.5
3.5	1.9 ^a^	3.6	3.8 ^a^	4.3	30.3 ^b^	5.8	22.0 ^b^	6.5
11	42.4 ^a^	2.4	42.5 ^a^	4.6	46.6 ^a^	1.4	44.5 ^a^	2.5
15	47 ^a^	1.7	46.9 ^a^	3.4	50.4 ^a^	1.9	48.4 ^a^	2.1
30	56.5 ^a^	3.4	55.9 ^a^	2.8	54.8 ^a^	2.8	55.3 ^a^	2.9
60	60 ^a^	3.2	61.4 ^a^	1.5	60.1 ^a^	3.3	59.1 ^a^	1.1

**Table 3:** Descriptive statistics (mean and standard deviation, SD) for the micro-mechanical properties as a function of depth and curing mode.

Parameter	Depth mm	Curing Mode			
		Self		Dual	
		Mean	SD	Mean	SD
Martens Hardness N/mm^2^	1	517.6	119.8	548.5	83.3
	2	537.3	94.9	561.9	93.9
	3	545.2	100.2	554.8	99.2
	4	548.1	95.1	553.2	99.1
	5	554.4	88.1	560.4	102.8
	6	552.3	92.1	548.2	90.4
Vickers Hardness N/mm^2^	1	64.7	15.7	69.1	11.1
	2	66.9	12.4	70.5	12.4
	3	68.1	12.8	69.2	12.8
	4	68.5	12.8	68.8	13.3
	5	69.0	11.8	69.0	11.1
	6	69.2	12.2	68.7	12.1
Indentation Modulus, GPa	1	13.6	3.1	14.3	2.1
	2	14.3	2.5	15.0	2.4
	3	14.6	2.6	14.8	2.4
	4	14.6	2.2	14.6	2.3
	5	14.9	2.1	14.9	1.8
	6	14.5	2.1	14.4	2.0

**Table 4:** Multivariate analysis (general linear model) assessing the effect of the parameter’s immersion time and medium on the measured properties (*p* < 0.05).

Properties	Parameter	*p*
Flexural strength	Time	0.618
	Medium	0.396
	Time × Medium	0.700
Flexural modulus	Time	0.593
	Medium	0.931
	Time × Medium	0.335
