# Erratum: Ilie, N. Comparative Effect of Self- or Dual-Curing on Polymerization Kinetics and Mechanical Properties in a Novel, Dental-Resin-Based Composite with Alkaline Filler. Running Title: Resin-Composites with Alkaline Fillers. *Materials* 2018, *11*, 108

**DOI:** 10.3390/ma13235547

**Published:** 2020-12-07

**Authors:** Nicoleta Ilie

**Affiliations:** Department of Conservative Dentistry and Periodontology, University Hospital, LMU Munich, Goethestr 70, 80336 Munich, Germany; nilie@dent.med.uni-muenchen.de; Tel.: +49-89-44005-9412; Fax: +49-89-44005-9302

**Incorrect Title**

There is an error in the title [1]. The correct title of the article is “Comparative Effect of Self- or Dual-Curing on Polymerization Kinetics and Mechanical Properties in a Novel, Dental-Resin-Based Composite with Alkaline Fille”. We apologize for this error and state that the scientific conclusions are unaffected. The original article has been updated. 
